# Perspectives on the Use of Mesenchymal Stem Cells in Vascularized Composite Allotransplantation

**DOI:** 10.3389/fimmu.2013.00175

**Published:** 2013-07-23

**Authors:** Jan A. Plock, Jonas T. Schnider, Mario G. Solari, Xin Xiao Zheng, Vijay S. Gorantla

**Affiliations:** ^1^Department of Plastic Surgery, University of Pittsburgh Medical Center, Pittsburgh, PA, USA; ^2^Division of Plastic and Hand Surgery, University Hospital Zurich, Zurich, Switzerland; ^3^Research Center for Translational Medicine, Shanghai East Hospital, Tongji University, Shanghai, China

**Keywords:** adipose stem cells, cell-based therapy, immunomodulation, composite tissue allotransplantation, tolerance

## Abstract

Reconstructive transplantation has emerged as clinical reality over the past decade. Long-term graft acceptance has been feasible in extremity and facial vascularized composite allotransplantation (VCA) under standard immunosuppression. Minimizing overall burden of lifelong immunosuppression is key to wider application of these non-life saving grafts. Allograft tolerance is the holy grail of many cell-based immunomodulatory strategies. Recent protocols using mesenchymal stem cells from bone marrow and adipose tissue offer promise and potential in VCA. This article provides an overview of the experimental basis, the scientific background and clinical applications of stem cell-based therapies in the field of reconstructive allotransplantation.

## Introduction

Complex reconstructions after major trauma are limited by available tissues, morbidity from extensive surgery, prolonged rehabilitation, suboptimal results, and costs of multiple surgeries. For such intricate injuries, vascularized composite allotransplantation (VCA) can achieve optimal restoration of tissue deficits with improved functional and esthetic results, enabling social and professional reintegration. During the past decade, more than 100 reconstructive transplant procedures have been performed around the world, including over 90 hand and 24 facial transplants with encouraging overall outcomes (1–3).

Like solid organ transplants, VCA requires long-term multidrug immunosuppression to prevent graft rejection mediated predominantly by the highly immunogenic skin component in these allografts. Medication toxicity could result in metabolic, infectious, or neoplastic complications. VCA is inherently different from solid organ transplantation in its non-life saving, yet life-enhancing impact on recipients. Further, unlike solid organs, clinical success is dictated not only by graft acceptance and survival, but also by nerve regeneration, which determines ultimate functional outcomes. These characteristics of VCA, drive the debate focused on the risks of lifelong immunosuppression mandated for graft survival balanced against the benefits of functional and quality of life outcomes. Thus, implementation of cellular therapies that integrate the concepts of immune regulation for graft acceptance with those of nerve regeneration could optimize the outcomes of these reconstructive modalities and minimize overall burden of immunosuppression. Such strategies could expand clinical feasibility and realize routine applicability of reconstructive transplantation.

Mesenchymal stem cells (MSCs) are pluripotent cells that are present in multiple tissues, including bone marrow (BM), adipose tissue, skin, muscle, blood, and placenta and can be isolated and expanded *ex vivo*. MSCs are capable of differentiation *in vitro* along multiple mesenchymal lineages such as osteocytes, chondrocytes, myocytes, adipocytes, and Schwann cells (SC) thereby emerging as a promising tool for tissue engineering and cell therapy.

Current literature on MSCs points to a wide range of immunological functions and interactions with other cell types (4, 5). Recent data support findings that MSCs mediate their actions through multiple mechanisms including paracrine effects. Various groups have shown some key differences between adipose-derived MSCs (AD-MSCs) and bone marrow derived MSCs (BM-MSCs) *in vitro* and *in vivo* (6–8). In particular, it has been demonstrated that MSCs have the capacity to suppress T cell activation and proliferation (9). Compared to BM, adipose tissue is a rich source of MSCs with up to 10-fold higher yield of MSCs (10). More recent publications demonstrate that AD-MSCs might also have higher immunomodulatory and immunosuppressive potential *in vitro* as compared to BM-MSCs (11–20). The ease of procurement of large volumes of AD-MSCs through techniques such as liposuction is an important benefit because of expeditious approach and minimal morbidity. Depending on time considerations for cell expansion, the overall duration of cell retrieval to cell infusion could be significantly shortened for AD-MSCs as compared to MSCs from other sources.

There is a growing complement of first in human studies addressing potential of MSC based cell therapies in autoimmune diseases, facilitation of hematopoietic stem cell engraftment in BM transplantation and in solid organ transplantation (4, 21, 22). Recent experimental and clinical studies highlight their potential for immunomodulation, tolerance induction, and prophylaxis and treatment of graft versus host disease (GvHD) (23–25). In VCA, the efficacy and effectiveness as well as mechanisms and outcomes of such therapies may be affected by the antigenicity of the skin component (26), as or by the differential tissue composition of these grafts.

## Immunological Function of MSCs

**Figure 1:**
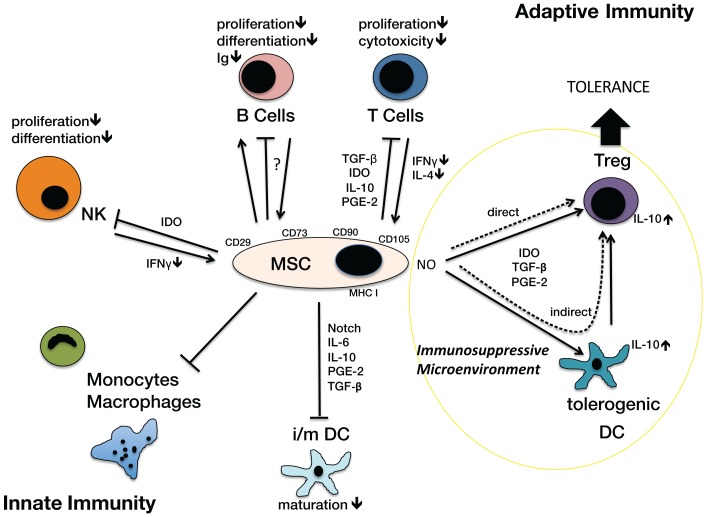
**Immunological Function of MSCs on different cell types of the innate and adaptive immunosystem**. MSC, mesenchymal stromal cell, characterized by surface antigens CD29, CD73, CD90 (CD105), MHC I; cell types: B cells, T Cells, Treg; regulatory T cells; DC, dendritic cells (tolerogenic, i/m, immature/mature), monocytes, macrophages, NK, natural killer cells. Arrows indicate activation or induction, T-bars indicate blockade of function or activation, in particular inhibition of proliferation, differentiation, cytotoxicity, maturation. The NO system is closely involved in creating an immunosuppressive microenvironment. Via an indirect way the generation of tolerogenic DCs induces Tregs. MSCs can also directly activate Treg generation. These Tregs play a significant role in the development of tolerance.

The effects of MSCs on innate and adaptive immunity have been reported in the literature (4, 5). MSCs modulate the innate function of monocytes, macrophages, natural killer (NK) cells, and dendritic cells (DCs). They are capable of modifying the maturation of DC, thereby inhibiting their antigen-presenting function and inducing the generation of tolerogenic DCs. This results in downregulated MHC II and chemokine expression (27–30). MSCs show intermediate expression of MHC I and do not express MHC II on their surface, which reduces their antigenicity. However the intracellular MHC II can become relevant when NK lyse transplanted MSCs (31, 32). In addition, MSCs also affect innate immunity through HLA-G expression leading to inhibition of NK cells and reduction of IFN-g expression (33, 34). English et al. (35) have shown that MSCs can directly induce regulatory T cell (Treg) generation. Mediators of Treg generation include indoleamine 2,3-dioxygenase (IDO), prostaglandin E2 (PGE2), and IFN-g (4). However, MSCs inhibit the activation of cytotoxic lymphocytes and immunoglobulin production through activated and proliferating B cells (36). The nitric oxide (NO) system as well as factors involved in stem cell recruitment like SDF-1, growth factors like VEGF and cytokines like IL-6, IL-8, and IL-10 have been shown to be important players in MSC dependent effects on adaptive immune responses (4, 5) (Figure 1).

## MSCs Promote Nerve Regeneration

The functional advantages of VCA over prosthetic fitting rely not only on motor recovery but also sensory recovery following regeneration of peripheral nerves and reintegration of neuronal pathways into the premotor cortex of the brain. MSCs have shown promise in improving clinical and electrophysiological outcomes in animal models of peripheral nerve injury (37–39). In comparison to local delivery, some groups have demonstrated significantly accelerated functional neuronal recovery following systemic administration of MSCs (40). The postulated mechanisms for such outcomes could include direct and paracrine effects (38, 41).

## MSC Use in Allotransplantation

Bartholomew et al. (9) first described prolongation of skin graft survival after allotransplantation in primates with a single application of expanded BM-MSCs at day 0. Sbano et al. (42) also reported similar results with cultured BM-MSC administered on day 0 and 3 in a rat model. Skin graft survival was prolonged, although grafts were rejected without supplementary immunosuppression.

Solari et al. (43) demonstrated long-term islet allograft survival in rats using syngeneic MSCs. In this study, repetitive application of syngeneic BM-MSCs was superior to allogeneic BM-MSCs. However, long-term tolerance could only be achieved in 40%, and cells were expanded up to P8. Their inherent immunomodulatory function has promoted use of MSCs in BM transplantation, solid organ transplantation, and treatment of GvHD (30). BM-MSCs have been used in experimental animal heart, liver, kidney, and islet transplantation (14, 30, 43–49). Immunomodulation was related to the suppression of alloreactive effector lymphocytes (50) through expression of a variety of cytokines (51–53) (Figure 1) and generation of CD4/CD25/FoxP3 Tregs that achieved long-term tolerance in animal studies via adaptive immune mechanisms (31, 50). Data on AD-MSCs in solid organ transplantation is scarce; however sources for MSCs other than BM have been supported/investigated (54). In addition, studies have confirmed the multifaceted properties of MSCs including their potential as part of induction therapy (55), in GvHD (56), tolerance induction (55, 57), and facilitating engraftment of BM transplants (58, 59). Several clinical trials have demonstrated their immunosuppressive function that could have potential in autoimmune disease (32, 60, 61). Such properties could offer potential for MSCs in acute and chronic rejection after VCA. MSCs have been successfully used in pediatric patients as rescue therapy of GVHD and repetitive rejection of BM transplants (62).

## Critical Issues for MSC Use in VCA

The success of organ transplantation and VCA is dependent on graft acceptance in the absence of GvHD. Various induction and maintenance regimens have been successful in controlling acute rejection. All these drugs have their role in VCA for specific immunosuppressive functions, but their unwarranted collateral effects on MSCs are less well investigated.

Several studies in solid organs have reported pre-transplant, peri-transplant, or post-transplant use of MSCs for immunomodulation (63–65). However, the negative effects of the depletion regimens that include irradiation, and polyclonal (antithymocyte globulin/serum), or monoclonal antibodies (e.g., alemtuzumab) on MSC recruitment, homing, and function remain to be clarified.

### Interaction with immunosuppressive drugs

Several immunosuppressive drugs significantly affect *in vitro* activity of MSCs. Hoogduijn et al. (66) investigated the *in vitro* effect of tacrolimus, rapamycin, and MPA on human MSCs and *vice versa*. Exposure of MSCs to high dosages of tacrolimus was toxic and reduced cell viability. While tacrolimus did not inhibit proliferation rates of MSCs, rapamycin, and MPA led to a dose-dependent reduction of proliferation and differentiation at therapeutic dosage levels. Unlike rapamycin, tacrolimus did not affect the osteogenic differentiation potential of MSCs (67). The effects of Cyclosporin A (CsA) on MSCs were insignificant. Some authors demonstrated a synergistic immunosuppressive effect of MSCs and MPA (68, 69). However, the immunomodulatory properties of MSCs were antagonized by rapamycin and tacrolimus. *In vivo* experiments support these findings of synergistic or opposing effects between MSC and pharmacological immunosuppression (68, 70). Hoogduijn’s et al (66) reported that MSCs inhibited the suppressive effect of tacrolimus and rapamycin on alloreactive lymphocytes but had no effect on MPA.

### Timing and dosage of MSCs

Currently, there is no consensus on dosing and timing of administration of MSCs in cell-based VCA protocols. Experimental studies indicate that the dosage of MSCs showing beneficial results ranges from 5 × 10^5^ to 5 × 10^7^ cells/kg body weight and time point (Table 1). In rats, the total amount of BM-MSCs administered over time was 6−10 × 10^6^ cells, whereas in pigs total amounts from 5 to 12.5 × 10^7^. BM-MSCs were used. In a study utilizing AD-MSCs, as many as 24 × 10^6^ cells/kg bodyweight were administered over three time points (58, 59, 71). MSCs were administered as early as day −30 to as late as +21 relative to transplantation or at different frequency in the interim (57–59). The time point was chosen with regards to the desired effect such as induction, immunomodulation, or support of BM engraftment.

**Table 1:** **Overview of the currently available experimental literature on MSC based cellular therapy for immunomodulation in VCA**.

Reference	Type of graft	Species	MSCs amount/type	MSC application	Induction regimen	Immuno-suppression	Main outcome
Pan et al. (59)	Hindlimb	Rat	10^7^ BM-MSCs (allogeneic)	Day −30	Irradiation, ALS, BMT	Rapamycin	Chimerism in peripheral blood
							Tolerance (>100 days)
							Protection against GvHD
Kuo et al. (58)	Hindlimb	Pig	10^7^ BM-MSCs (allogeneic)	Day −1, +3, +7, +14, +21	Irradiation, BMT	Cyclosporin	Perivascular MSC engraftment (graft)
							Chimerism in peripheral blood
							Tolerance (>200 days)
							Protection against GvHD
							Treg ⇑
Kuo et al. (87)	Hindlimb	Pig	10^7^ BM-MSCs (allogeneic)	Day −1, +3, +7, +14, +21	Irradiation	Cyclosporin	Tolerance (>120 days)
							Treg ⇑
Kuo et al. (57)	Hindlimb	Rat	2 × 10^6^ AD-MSCs (allogeneic)	Day +7, +14, +21	ALS	Cyclosporin	Tolerance (>150 days)
							Chimerism peripheral blood
							Treg ⇑
							IL-10 ⇑
Kuo et al. (88)	Face	Pig	2.5 × 10^7^ BM-MSCs (allogeneic)	Day −1, +3, +7, +14, +21	N/A	Cyclosporin	Treg ⇑
							IL-10 ⇑
Aksu et al. (71)	Skin flap	Rat	2–3 × 10^6^ (repetitive) BM-MSCs (syngeneic)	Days 0, +7, +14, and +21	Irradiation BMT (repetitive)	Cyclosporin	Syngeneic MSCs limit toxicity of allogeneic BMT
							Prolongation of tolerance
							Enhanced mixed chimerism

### Homing of MSCs/chimerism

Eggenhofer et al. (72) recently reported a murine study reduced life span of intravenously delivered cultured MSCs due to entrapment in the lung capillaries or liver sinusoids. Others have shown distribution to other organs like kidney and spleen, BM, and peripheral blood (73). Kuo et al. (58) demonstrated recruitment and homing of MSCs to perivascular sites with long-term survival in VCA models. In addition peripheral blood chimerism after VCA with MSC-co-transplantation was demonstrated in pig and rat models (57–59). Indeed, the first-pass clearance of MSCs in lung and liver may be an obstacle for these cell therapies (74, 75). Several strategies including vasodilation, co-transplantation, and repetitive infusions have been suggested to increase cell passage. Freshly isolated MSCs have been shown to be superior to culture-expanded MSCs in terms of lower entrapment potential due to smaller size as well as better homing potential (76). They are found in high number in sites of trauma and ischemia after several days (77).

### Cultured MSCs

For therapeutic indications, MSCs need to be expanded in culture to achieve sufficient numbers for transplantation. Karp and Leng (78) reported that the culture medium and conditions are likely to influence the properties of MSCs as well as their morphology (79, 80). The number of passages inversely correlates with the number of surface antigens and homing potential (81–84). Cultured MSCs may be morphologically indistinguishable from fibroblasts and even show similar cell-surface markers, differentiation potential, and immunologic properties (80). Multiple cell passages not only affect proliferation and differentiation but also alter cell-surface antigens and cytokine production (85, 86). To our knowledge, there is no study to date on the immunomodulatory potential of long-term-cultured MSCs. In our view, short-term culture of MSCs (<P3) and freshly isolated MSCs must be given preference in cell therapy protocols until we gain more insights into the properties of expanded MSCs.

## MSCs in Vascularized Composite Allotransplantation

Only a few groups have advocated the use of BM-MSCs or AD-MSCs for immunomodulatory strategies in VCA (Table 1). Despite growing enthusiasm, the basic experience is limited to few experimental studies with MSCs. Aksu et al. (71) reported that co-administration of host BM-MSCs with unmodified donor BM and immunosuppression (CsA + irradiation) enabled prolonged survival of full MHC mismatched rodent vascularized skin grafts with generation of mixed chimerism and absence of GvHD. Outcomes positively correlated with number of times the BM-MSCs were administered. Pan et al. (59) reported a rat hindlimb VCA model where limb transplants were performed a month after conditioning with total body irradiation, and anti-lymphocyte-serum followed by allogeneic BM-MSC and BM infusion. This resulted in stable chimerism, donor specific tolerance, and no GvHD. Allogeneic BM-MSC transplantation with or without co-transplantation of BM has been shown to be successful in prolongation (>200 days) of pig limb allograft survival after irradiation and CsA treatment (58, 87). Repetitive high dose BM-MSC treatment was also successful in prolonging survival in a pig hemi-facial transplantation model without conditioning therapy (88). The authors reported only mild rejection of the graft (Grade I–II), improved under CsA treatment. The positive effects of BM-MSCs on rejection grades were correlated to IL-10 upregulation and Treg induction. Despite the prolongation, all grafts succumbed within 90 days. In a different approach, Kuo et al. (57) administered three fold numbers of AD-MSCs under temporary immunosuppression in a rat hindlimb allotransplantation model. After cessation of immunosuppression, this regimen prolonged allograft survival significantly with stable tolerance in 89% for>150 days. Treg populations were significantly increased and elevated donor lymphoid cell counts (RT1n) resulted in stable peripheral blood chimerism until endpoint. The same group conducted a study in a swine hindlimb allotransplantation model using BM-MSCs, irradiation, and short-term CsA. They were able to demonstrate prolongation of the survival (>100 days in 67%) and an increase of the Treg population.

## Conclusion

Taken together, emerging literature evidence highlights the potential promise of MSC based cellular therapies for immunomodulation and neurodegeneration in VCA. Extensive experimental and clinical studies in the areas of solid organ transplantation, hematopoietic stem cell co-transplantation and autoimmune disease underscore the relevance and impact of MSC based therapies in VCA. Traditionally, the chief obstacle hampering application of BM-MSCs has been the limited cell yield and requirement for donor cell expansion. The high cell yields of AD-MSCs from adipose sources obtained through easy, fairly non-invasive techniques have enabled expeditious cell processing, thus expanding the clinical feasibility of these therapies. These advantages, combined with the insights supporting the superior immunomodulatory potential of AD-MSCs versus BM-MSCs truly advocate adipose-based cellular therapies. The higher cell yields also facilitate repetitive infusion both systemically and locally in the graft. Freshly isolated AD-MSCs can overcome the loss of viability and entrapment during the first-pass phenomenon in the capillary systems of the lung. Importantly, MSCs mediate paracrine effects on remote tissues through specific cytokines, chemokines, and growth factors. It still remains to be defined if such paracrine effects mediate also tolerogenic or immunomodulatory effects in VCA or other applications. Future protocols should carefully address the dosing and timing of MSC administration and the effects of conditioning regimens and maintenance immunosuppression on function of these cells. Most notably, the effect of MSC based strategies on nerve regeneration, critical for functional outcomes in VCA offers an uncharted area of investigation. Thus far, only one donor BM cell-based protocol has been clinically evaluated in human upper extremity VCA (Pittsburgh Protocol) and involves megadose donor derived BM infusion after at day 11–14 after surgery (89). Insights into the impact of such protocols in minimizing the need for dosing, frequency, and duration of immunosuppression are just emerging. Meanwhile, we must continue to explore the multifaceted potential of MSCs in experimental VCA to further fine tune standard protocols as used in conventional VCA or solid organ transplantation. The true clinical scope and impact of disparate VCA strategies will only be realized when such protocols enable optimization of the functional and immunological benefits of these novel reconstructive procedures while reducing long-term risk of lifelong immunosuppression.

## Conflict of Interest Statement

The authors declare that the research was conducted in the absence of any commercial or financial relationships that could be construed as a potential conflict of interest.
